# Stress hyperglycemia ratio is associated with systemic inflammation and clinical outcomes in diabetic inpatients with pneumonia on admission

**DOI:** 10.1111/1753-0407.13398

**Published:** 2023-05-05

**Authors:** Bing Liu, Yu Chen, Liping Yu, Min Zhou

**Affiliations:** ^1^ Department of Pulmonary and Critical Care Medicine, Ruijin Hospital Shanghai Jiao Tong University School of Medicine Shanghai China; ^2^ Institute of Respiratory Diseases Shanghai Jiao Tong University School of Medicine Shanghai China; ^3^ Shanghai Key Laboratory of Emergency Prevention, Diagnosis and Treatment of Respiratory Infectious Diseases Shanghai China; ^4^ Department of Pulmonary and Critical Care Medicine Shengjing Hospital of China Medical University Shenyang China; ^5^ Department of Endocrinology China‐Japan Friendship Hospital Beijing China

**Keywords:** clinical outcomes, diabetic inpatients, pneumonia, stress hyperglycemia ratio, systemic inflammation, 临床结局, 糖尿病住院患者, 肺炎, 应激性高血糖比值, 系统性炎症

## Abstract

**Backgrounds:**

Stress hyperglycemia ratio (SHR) reflects the acute blood glucose change in response to acute illnesses or injuries, including pneumonia. We aimed to investigate the associations of SHR with systemic inflammation and clinical outcomes in diabetic inpatients with pneumonia on admission.

**Methods:**

A multicenter and retrospective study was conducted among diabetic inpatients with pneumonia on admission via electronic medical records from 2013 to 2019 in Ruijin Hospital, Shengjing Hospital, and China‐Japan Friendship Hospital.

**Results:**

The study included 1631 diabetic inpatients with pneumonia on admission. Patients of the fourth quartile (Q4) of SHR on admission showed significantly elevated systemic inflammation compared with those of the first quartile (Q1), second quartile (Q2), or third quartile (Q3) of SHR, including more white blood cells (9.1 × 10^9^/L in Q4 vs 7.6 × 10^9^/L in Q1, 7.9 × 10^9^/L in Q2, and 8.0 × 10^9^/L in Q3, *p* < .001), higher neutrophil‐to‐lymphocyte ratio (7.0 in Q4 vs 3.6 in Q1, 3.8 in Q2, and 4.0 in Q3, *p* < .001), higher C‐reactive protein (52.8 mg/L in Q4 vs 18.9 mg/L in Q1, *p* < .001; 52.8 mg/L in Q4 vs 28.6 mg/L in Q2, *p* = .002), higher procalcitonin (0.22 ng/mL in Q4 vs 0.10 ng/mL in Q1, 0.09 ng/mL in Q2, and 0.11 ng/mL in Q3, *p* < .001), and higher D‐dimer (0.67 mg/L in Q4 vs 0.47 mg/L in Q1, 0.50 mg/L in Q2, and 0.47 mg/L in Q3, *p* < .001). Excluding patients with hypoglycemia on admission in the analyses, there were still distinct J‐shaped associations between SHR and adverse clinical outcomes in patients with different severity of pneumonia, especially in those with CURB‐65 score for pneumonia severity (Confusion, blood Urea nitrogen, Respiratory rate, Blood pressure) ≥ 2. In the multivariable regression model, predictive value for adverse clinical outcomes was higher when SHR was taken as a spline term than as quartiles in all patients (area under curve 0.831 vs 0.822, *p* = .040), and when SHR as a spline term instead of fasting blood glucose was included in patients with CURB‐65 ≥ 2 (area under curve 0.755 vs 0.722, *p* = .027).

**Conclusions:**

SHR was correlated with systematic inflammation and of J‐shaped associations with adverse clinical outcomes in diabetic inpatients with pneumonia of different severity. The inclusion of SHR in the blood glucose management of diabetic inpatients may be beneficial, especially for the prevention of potential hypoglycemia or the recognition of relative glucose insufficiency in those with severe pneumonia or high hemoglobin A_1C_.

## Graphical abstract

**Highlights**

- High stress hyperglycemia ratio (SHR) was associated with elevated systematic inflammation in diabetic inpatients with pneumonia on admission.
- In the absence of hypoglycemia on admission, SHR was of J‐shaped associations with adverse clinical outcomes in diabetic inpatients with pneumonia of different severity.
- In diabetic inpatients with pneumonia on admission, predictive value of SHR for adverse clinical outcomes was greater than that of fasting blood glucose and was further elevated when the nonlinear association was taken into consideration.

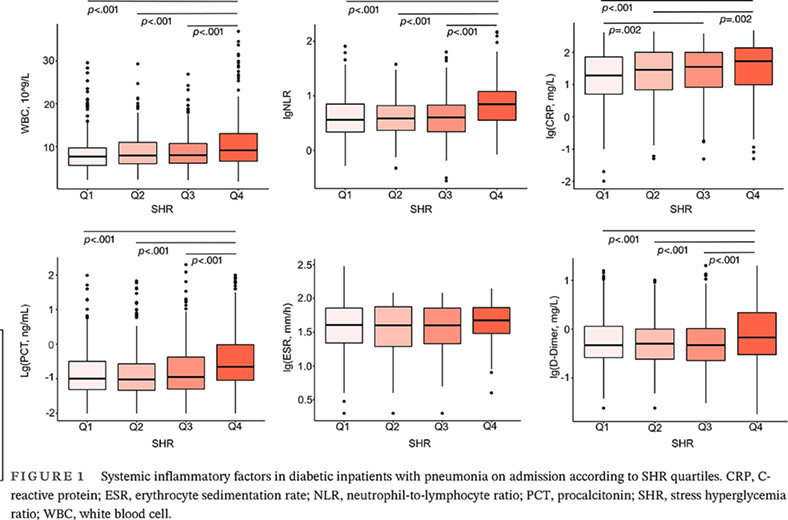

## 摘要

研究背景:应激性高血糖比率(Stress hyperglycemia ratio, SHR)反映急性疾病或损伤(包括肺炎)引起的急性血糖变化。本研究旨在探讨SHR与入院时合并肺炎的糖尿病住院患者系统性炎症和临床结局的相关性。

方法:采用多中心回顾性研究，通过电子病历收集2013至2019年在瑞金医院, 盛京医院和中日友好医院住院的糖尿病合并肺炎患者。

结果:本研究共纳入1631例入院时合并肺炎的糖尿病住院患者。入院时SHR处于第四四分位数(Q4)的患者与处于第一四分位数(Q1), 第二四分位数(Q2)或第三四分位数(Q3)的患者相比，显示出系统性炎症明显升高，包括更高的白细胞计数(Q4中为9.1x10^9^/L, Q1中为7.6x10^9^/L，Q2中为7.9x10^9^/L，Q3中为8.0x10^9^/L，P<0.001), 更高的中性粒细胞与淋巴细胞比值(Q4中为7.0, Q1中为3.6，Q2中为3.8，Q3中为4.0，P<0.001), 更高的C‐反应蛋白(Q4中为52.8 mg/L，Q1中为18.9 mg/L，P<0.001；Q4中为52.8 mg/L，Q2中为28.6 mg/L，P=0.002), 更高的降钙素原(Q4中为0.22 ng/mL，Q1中为0.10 ng/mL，Q2中为0.09 ng/mL，Q3中为0.11 ng/mL，P<0.001)以及更高的D‐二聚体(Q4中为0.67 mg/L，Q1中为0.47 mg/L，Q2中为0.50 mg/L，Q3中为0.47 mg/L，P<0.001)。在分析中排除入院时低血糖的患者后，在不同肺炎严重程度的患者中仍存在明显的J形关联，特别是在CURB‐65≥2的患者中。在多变量回归模型中，将SHR作为样条项而非四分位数时，对于所有患者的不良临床结果的预测价值更高(曲线下面积为0.831 vs. 0.822，P=0.040)，并且在CURB‐65≥2的患者中，当将SHR作为样条项代替空腹血糖时，预测不良临床结果的能力也更高(曲线下面积为0.755 vs. 0.722，P=0.027)。

结论:SHR与不同严重程度糖尿病合并肺炎住院患者的系统性炎症相关，且与不良临床结局呈J形关系。将SHR纳入住院糖尿病患者的血糖管理可能是有益的，特别是对于预防潜在的低血糖或识别重症肺炎或高糖化血红蛋白的相对葡萄糖不足。

## 1 INTRODUCTION

Pneumonia and diabetes are both among the top 10 causes of death globally according to the World Health Organization.^1^ It was reported by the International Diabetes Federation that 537 million adults are currently living with diabetes worldwide, accounting for 10.5% of the total adult population.^2^ With altered immune response and disturbed airway glucose homeostasis, patients with diabetes are proved to have higher morbidity, severity, and mortality of pneumonia.^3^, ^4^ In diabetic individuals suffered from pneumonia, management of blood glucose is of crucial importance.^5^ Several international guidelines have been developed to guide the management of blood glucose in diabetes to avoid hyperglycemia and hypoglycemia.^5^, ^6^, ^7^, ^8^, ^9^ However, stress hyperglycemia is common in pneumonia, acting as a physiological response to acute illnesses or injuries.^10^ In case of pneumonia, blood glucose would reflect a composite glucose level of stress blood glucose and background blood glucose. Unlike patients without diabetes, patients with diabetes are of largely varied background glucose levels. Previous studies also indicated that the association between blood glucose, both hyperglycemia and hypoglycemia, and clinical outcomes was altered by former blood glucose level.^11^, ^12^, ^13^

Recently, several studies highlighted the value of stress hyperglycemia, which presents the absolute glycemia change, in the analyses of prognoses in acute physiological events including pneumonia.^13^, ^14^, ^15^, ^16^, ^17^, ^18^, ^19^, ^20^, ^21^ In these studies, new metrics, including stress hyperglycemia ratio (SHR), glycemic gap, or glucose concentration‐to‐hemoglobin A_1C_ ratio (GAR), were used to represent stress hyperglycemia. Among previous studies, much attention was paid to the hazard of severe stress hyperglycemia to clinical outcomes but little was paid to the relative deficiency of stress blood glucose. In addition, management of blood glucose in patients with and without diabetes, in patients from different medical settings, and in patients with different severity of illness is not the same.^5^, ^6^, ^7^, ^8^, ^9^ Thus far, studies focused on stress hyperglycemia on diabetic patients with pneumonia on admission were limited and of small sample size. Also, systemic inflammatory status in patients with different stress hyperglycemia level needs further study. Here, we carried out a multicenter study with 1631 diabetic inpatients to explore the association of SHR with systemic inflammation and clinical outcomes in patients with pneumonia of different severity by both linear and non‐linear analyses.

## 2 METHODS

### 2.1 Study participants and data collection

The study was a multicenter, descriptive study conducted from 2013 to 2019 in Ruijin Hospital, Shengjing Hospital, and China‐Japan Friendship Hospital. Data on demographic information, laboratory tests on admission, treatments, and outcomes were extracted and collected retrospectively from medical records. Patients were included in the study according to the following criteria: (1) aged no less than 18 years old; (2) pneumonia on admission; (3) diabetes; (4) available fasting blood glucose (FBG) and hemoglobin A_1C_ (HbA_1C_), for the calculating of SHR, and CURB‐65 (Confusion, blood Urea nitrogen, Respiratory rate, Blood pressure) scores on admission; (5) complete demographic information (including age, sex, and comorbidities); and (6) definite clinical outcomes (including need for mechanical ventilation and in‐hospital mortality). Exclusion criteria were as follows: (1) absolute hypoglycemia on admission (FBG ≤ 3.9 mmol/L); and (2) current pulmonary tuberculosis. Totally, 1631 cases were included in the analyses (975 in Shengjing Hospital from 2013 to 2019, 520 in Ruijin Hospital from 2017 to 2019; and 136 in China‐Japan Friendship Hospital from 2017 to 2019) (Figure S1). The ethics committees of local hospitals approved this study and waived the need for written informed consents.

### 2.2 Definitions

The diagnosis of diabetes was based on American Diabetes Association.^22^ Pneumonia was defined according to Centers for Disease Control and Prevention.^23^ SHR was measured by FBG on admission divided by estimated average chronic glucose (eAG).^24^ The eAG was calculated based on HbA_1C_ according to the published formula e*AG* (mg/dL) = 28.7 × HbA_1C_ (%) − 46.7.^25^ In the analyses of adverse clinical outcomes, we used composite end point, which included need for mechanical ventilation at hospitalization or in‐hospital mortality. Severity of pneumonia was roughly classified by CURB‐65 scores on admission.

### 2.3 Statistical analyses

Continuous variables were presented as range or median (interquartile range). Categorical variables were shown as count (percentage). Comparisons among groups for continuous variables were performed using the Kruskal–Wallis H‐test and further pairwise comparisons were done using Wilcoxon rank‐sum test. Chi‐square test with pairwiseNominalIndependence function in R package *rcompanion* were employed in group comparisons for categorical variables with *p* values adjusted by Bonferroni correction in the intragroup comparisons. Associations between SHR on admission and composite end point were explored using univariable and multivariable logistic regression analyses, and the results were listed as odds ratio (OR) with 95% confidence interval (CI). Restricted cubic splines based on logistic regressions were used to examine the potential nonlinear associations between SHR and composite clinical outcome. Considering the sample size of this study, five knots automatically located by R in certain percentiles were used in the spline analyses. Predictive values of SHR in different statistical forms were evaluated and compared by area under curve (AUC) of receiver operating characteristic (ROC) using R package *pROC*. Two‐sided significance level of 0.05 was selected for all analyses. Statistics were analyzed and displayed using R version 4.2.0 (R Foundation for Statistical Computing).

## 3 RESULTS

### 3.1 Basic characteristics

In this study, 1631 diabetic inpatients with pneumonia on admission were included between January 2013 and December 2019; 1579 (96.8%) patients had previous diagnoses of diabetes, most of which were type 2 diabetes in those with recorded type of diabetes (1567/1579, 99.4%). The other 52 (3.2%) patients were of newly diagnosed diabetes. SHR on admission ranged from 0.17 to 3.12 and was divided into quartiles for the further analyses. Basic characteristics of patients were summarized an compared among patients of the first quartile (Q1), the second quartile (Q2), the third quartile (Q3), and the fourth quartile (Q4) of SHR, as shown in Table 1. Patients in Q4 were of higher CURB‐65 scores on admission (*p* < .001), longer length of hospital stay (13.0 days in Q4 vs 11.0 days in Q2, *p* = .011; 13.0 days in Q4 vs 11.0 days in Q3, *p* = .035), higher mechanical ventilation rate (13.0% in Q4 vs 5.2% in Q1, *p* < .001; 13.0% in Q4 vs 3.4% in Q2, *p* < .001; 13.0% in Q4 vs 6.4% in Q3, *p* = .013), and higher in‐hospital mortality (16.9% in Q4 vs 3.7% in Q1, *p* < .001; 16.9% in Q4 vs 2.5% in Q2, *p* < .001; 16.9% in Q4 vs 5.9% in Q3, *p* < .001). Thus, more patients in Q4 reached the composite end point compared with the other three groups (21.8% in Q4 vs 7.6% in Q1, *p* < .001; 21.8% in Q4 vs 4.9% in Q2, *p* < .001; 21.8% in Q4 vs 9.1% in Q3, *p* < .001).

**TABLE 1:** Basic clinical characteristics of diabetic inpatients with pneumonia on admission according to SHR quartiles.

Variable	Number of patients	Q1 (*n* = 407)	Q2 (*n* = 408)	Q3 (*n* = 408)	Q4 (*n* = 408)	*p* value
SHR (range)	1631	0.165–0.742	0.742–0.889	0.889–1.092	1.092–3.123	
Age (years)	1631	68.0 (60.0–78.0)	67.0 (58.0–77.0)	65.0 (58.0–76.0)	69.0 (59.0–79.0)	.055
Male	1631	224 (55.0)	249 (61.0)	253 (62.0)	253 (62.0)	.125
Body mass index	1108					.210
<18.5		19 (6.7)	11 (3.8)	21 (7.3)	14 (5.6)	
18.5–25.0		168 (59.4)	148 (51.6)	145 (50.2)	133 (53.4)	
25.0–30.0		76 (26.9)	97 (33.8)	97 (33.6)	84 (33.7)	
≥30.0		20 (7.1)	31 (10.8)	26 (9.0)	18 (7.2)	
Current smokers	1631	67 (16.5)	79 (19.4)	88 (21.6)	78 (19.1)	.327
Comorbidities	1631					
Chronic lung diseases		73 (17.9)	67 (16.4)	60 (14.7)	56 (13.7)	.361
Coronary heart disease		100 (24.6)	94 (23.0)	85 (20.8)	90 (22.1)	.628
Hypertension		232 (57.0)	203 (49.8)	213 (52.2)	232 (56.9)	.097
Stroke^c^ ^,^ ^e^ ^,^ ^f^		80 (19.7)	83 (20.3)	91 (22.3)	130 (31.9)	<.001
Chronic kidney disfunction		42 (10.3)	47 (11.5)	36 (8.8)	52 (12.7)	.314
Cirrhosis		4 (1.0)	7 (1.7)	10 (2.5)	14 (3.4)	.095
Rheumatic disease		16 (3.9)	9 (2.2)	6 (1.5)	14 (3.4)	.124
Malignancy		25 (6.1)	27 (6.6)	31 (7.6)	21 (5.1)	.546
FBG (mmol/L)^a^ ^,^ ^b^ ^,^ ^c^ ^,^ ^d^ ^,^ ^e^ ^,^ ^f^	1631	6.1 (5.2–7.7)	7.4 (6.4–8.9)	9.0 (7.6–11.0)	12.5 (9.9–15.6)	<.001
HbA_1C_ (%)^a^ ^,^ ^b^ ^,^ ^c^	1631	7.9 (6.9–9.5)	7.3 (6.6–8.6)	7.3 (6.5–8.6)	7.3 (6.5–8.5)	<.001
Course of diabetes (month)^c^ ^,^ ^e^ ^,^ ^f^	1545					<.001
<1		81 (20.5)	70 (18.1)	67 (17.6)	52 (13.6)	
1–12		29 (7.3)	31 (8.0)	25 (6.6)	25 (6.5)	
12–120		193 (48.7)	197 (51.0)	209 (55.0)	156 (40.7)	
≥120		93 (23.5)	88 (22.8)	79 (20.8)	150 (39.2)	
Treatment of diabetes^a^ ^,^ ^e^ ^,^ ^f^	1558					<.001
No		95 (24.2)	107 (27.3)	97 (25.3)	86 (22.1)	
Oral drugs only		147 (37.4)	175 (44.6)	161 (41.9)	126 (32.4)	
Insulin with/without oral drugs		151 (38.4)	110 (28.1)	126 (32.8)	177 (45.5)	
Chronic complications of diabetes	1079	33 (13.1)	41 (14.9)	55 (19.3)	48 (17.9)	.206
CURB‐65 scores on admission^c^ ^,^ ^e^ ^,^ ^f^	1631					<.001
0–1		319 (78.4)	318 (77.9)	306 (75.0)	236 (57.8)	
2		69 (17.0)	68 (16.7)	82 (20.1)	113 (27.7)	
3–5		19 (4.7)	22 (5.4)	20 (4.9)	59 (14.5)	
Length of hospital stay (days)^e^ ^,^ ^f^	1611	11.0 (8.5–15.0)	11.0 (8.0–15.0)	11.0 (9.0–15.0)	13.0 (9.0–18.0)	.007
Mechanical ventilation at hospitalization^c^ ^,^ ^e^ ^,^ ^f^	1631	21 (5.2)	14 (3.4)	26 (6.4)	53 (13.0)	<.001
In‐hospital mortality^c^ ^,^ ^e^ ^,^ ^f^	1631	15 (3.7)	10 (2.5)	24 (5.9)	69 (16.9)	<.001
Composite adverse clinical outcomes^c^ ^,^ ^e^ ^,^ ^f^	1631	31 (7.6)	20 (4.9)	37 (9.1)	89 (21.8)	<.001

*Note*: Comparisons for continuous variables were performed using the Kruskal‐Wallis H‐test and further pairwise comparisons between groups were done using Wilcoxon rank sum test. Comparisons for categorical variables were explored by chi square test with *p* values adjusted by bonferroni correction in the intra‐group comparisons.

Abbreviations: CURB‐65, score for pneumonia severity (Confusion, blood Urea nitrogen, Respiratory rate, Blood pressure); FBG, fasting blood glucose; HbA_1C_, hemoglobin A_1C_; Q1, the first quartile; Q2, the second quartile; Q3, the third quartile; Q4, the fourth quartile; SHR, stress hyperglycemia ratio.

^a^ *p* < .05 between Q1 and Q2.

^b^ *p* < .05 between Q1 and Q3.

^c^ *p* < .05 between Q1 and Q4.

^d^ *p* < .05 between Q2 and Q3.

^e^ *p* < .05 between Q2 and Q4.

^f^ *p* < .05 between Q3 and Q4.

### 3.2 Systemic inflammatory factors

**FIGURE 1:**
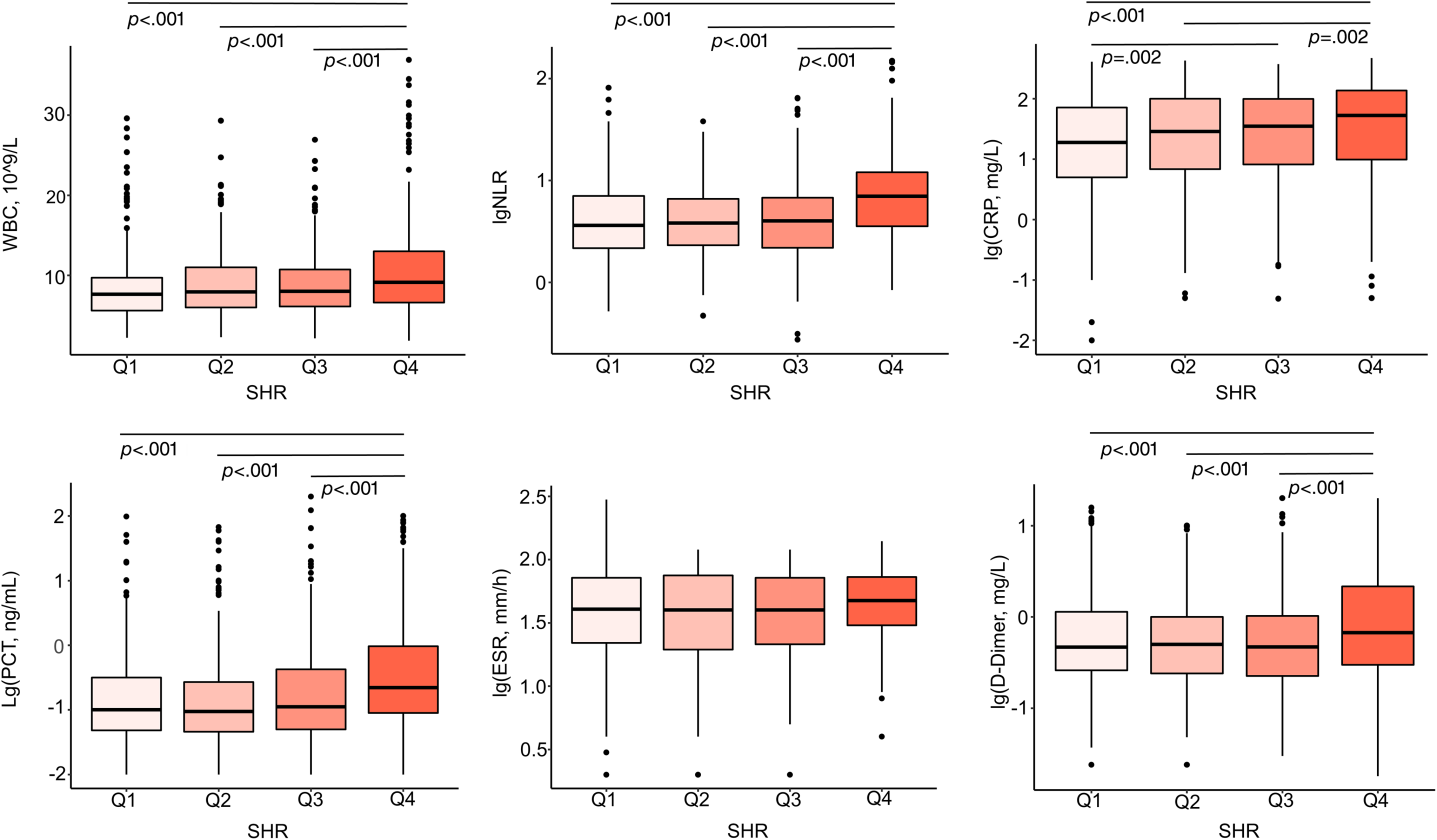
Systemic inflammatory factors in diabetic inpatients with pneumonia on admission according to SHR quartiles. CRP, C‐reactive protein; ESR, erythrocyte sedimentation rate; NLR, neutrophil‐to‐lymphocyte ratio; PCT, procalcitonin; SHR, stress hyperglycemia ratio; WBC, white blood cell.

As shown in Figure 1, six systemic inflammatory factors, including white blood cell count (WBC), neutrophil‐to‐lymphocyte ratio (NLR), C‐reactive protein (CRP), procalcitonin (PCT), erythrocyte sedimentation rate (ESR), and D‐dimer, were chosen to assess inflammatory levels in different SHR quartiles.^26^ In total, 1631 data on WBC, 1613 on NLR, 1496 on CRP, 1143 on PCT, 434 on ESR, and 1444 on d‐Dimer were available for these patients. Generally, compared with patients in other quartiles, patients in Q4 showed significantly elevated systemic inflammatory level with more WBC (9.1 × 10^9^/L in Q4 vs 7.6 × 10^9^/L in Q1, 7.9 × 10^9^/L in Q2, and 8.0 × 10^9^/L in Q3, *p* < .001), higher NLR (7.0 in Q4 vs 3.6 in Q1, 3.8 in Q2, and 4.0 in Q3, *p* < .001), higher CRP (52.8 mg/L in Q4 vs 18.9 mg/L in Q1, *p* < 0.001; 52.8 mg/L in Q4 vs 28.6 mg/L in Q2, *p* = .002), higher PCT (0.22 ng/mL in Q4 vs 0.10 ng/mL in Q1, 0.09 ng/mL in Q2, and 0.11 ng/mL in Q3, *p* < .001), and higher D‐Dimer (0.67 mg/L in Q4 vs 0.47 mg/L in Q1, 0.50 mg/L in Q2, and 0.47 mg/L in Q3, *p* < .001). No significant differences in ESR level were found among groups.

### 3.3 Associations between SHR quartiles and adverse clinical outcomes

**FIGURE 2:**
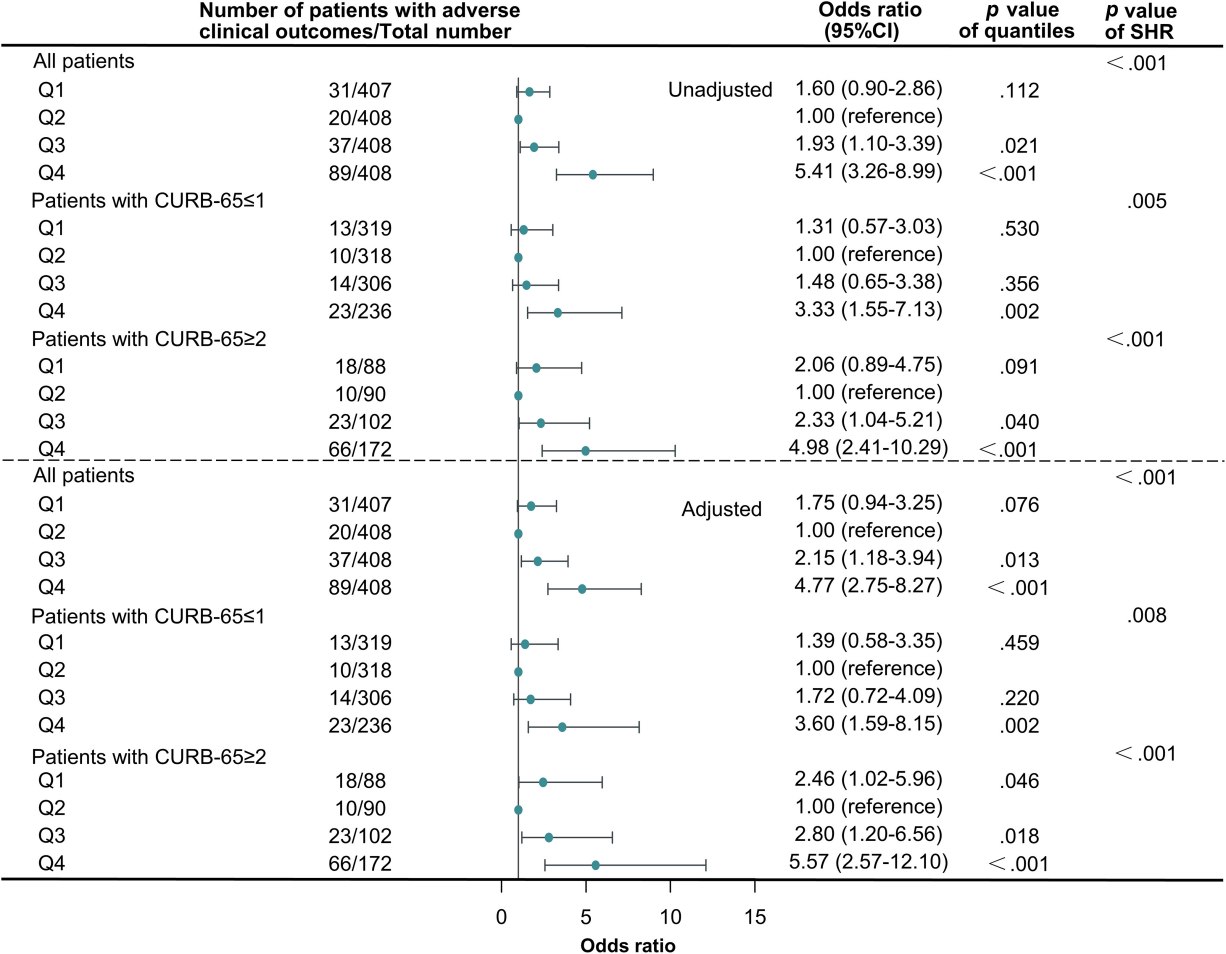
Forest plots showing unadjusted and adjusted odds ratios of SHR for adverse clinical outcomes in diabetic inpatients with pneumonia on admission. Odds ratios were analyzed in all patients, patients with CURB‐65 ≤ 1, and patients with CURB‐65 ≥ 2 separately by univariable and multivariable logistic regression analyses. In the multivariable logistic regression analyses, odds ratios were adjusted by age, sex, comorbidities (including chronic lung diseases, coronary heart disease, hypertension, stroke, chronic kidney disfunction, cirrhosis, rheumatic disease, and malignancy), current smoking status, and CURB‐65 scores on admission (as a continuous variable). CURB‐65, score for pneumonia severity (Confusion, blood Urea nitrogen, Respiratory rate, Blood pressure; Q1, the first quartile; Q2, the second quartile; Q3, the third quartile; Q4, the fourth quartile; SHR, stress hyperglycemia ratio.

Univariable and multivariable logistic regression analyses were done to determine the association between SHR and adverse clinical outcomes in all patients and patients of different severity classified by CURB‐65 scores (Figure 2). In the univariable logistic models, in which only SHR was included as a category variable, SHR was shown to be significantly associated with the composite clinical outcome in all patients and patients with different severity (*p* < .001). In the multivariable logistic regression models, we adjusted ORs of SHR by age, sex, chronic comorbidities (including chronic lung diseases, coronary heart disease, hypertension, stroke, chronic kidney disfunction, cirrhosis, rheumatic disease, and malignancy), current smoking status, and CURB‐65 scores. As shown in Figure 2, SHR was still a significant predictor for composite end point in all group sets (*p* < .001). Taken Q2 as a reference, patients in Q3 and Q4 were of higher risk of developing adverse clinical outcomes (OR: 2.15, 95% CI: 1.18–3.94 for Q3 in all patients, *p* = .013; OR: 4.77, 95% CI: 2.75–8.27 for Q4 in all patients, *p* < .001; OR: 3.60, 95% CI: 1.59–8.15 for Q4 in patients with CURB‐65 ≤ 1, *p* = .002; OR: 2.80, 95% CI:1.20–6.56 for Q3 in patients with CURB‐65 ≥ 2, *p* = .018; OR: 5.57, 95% CI: 2.57–12.10 for Q4 in patients with CURB‐65 ≥ 2, *p* < .001). What is more, in patients with CURB‐65 ≥ 2, Q1 possessed a slightly but significantly increased risk over Q2 (OR: 2.46, 95% CI: 1.02–5.96, *p* = .046).

### 3.4 Nonlinear correlations between SHR and adverse clinical outcomes

**FIGURE 3:**
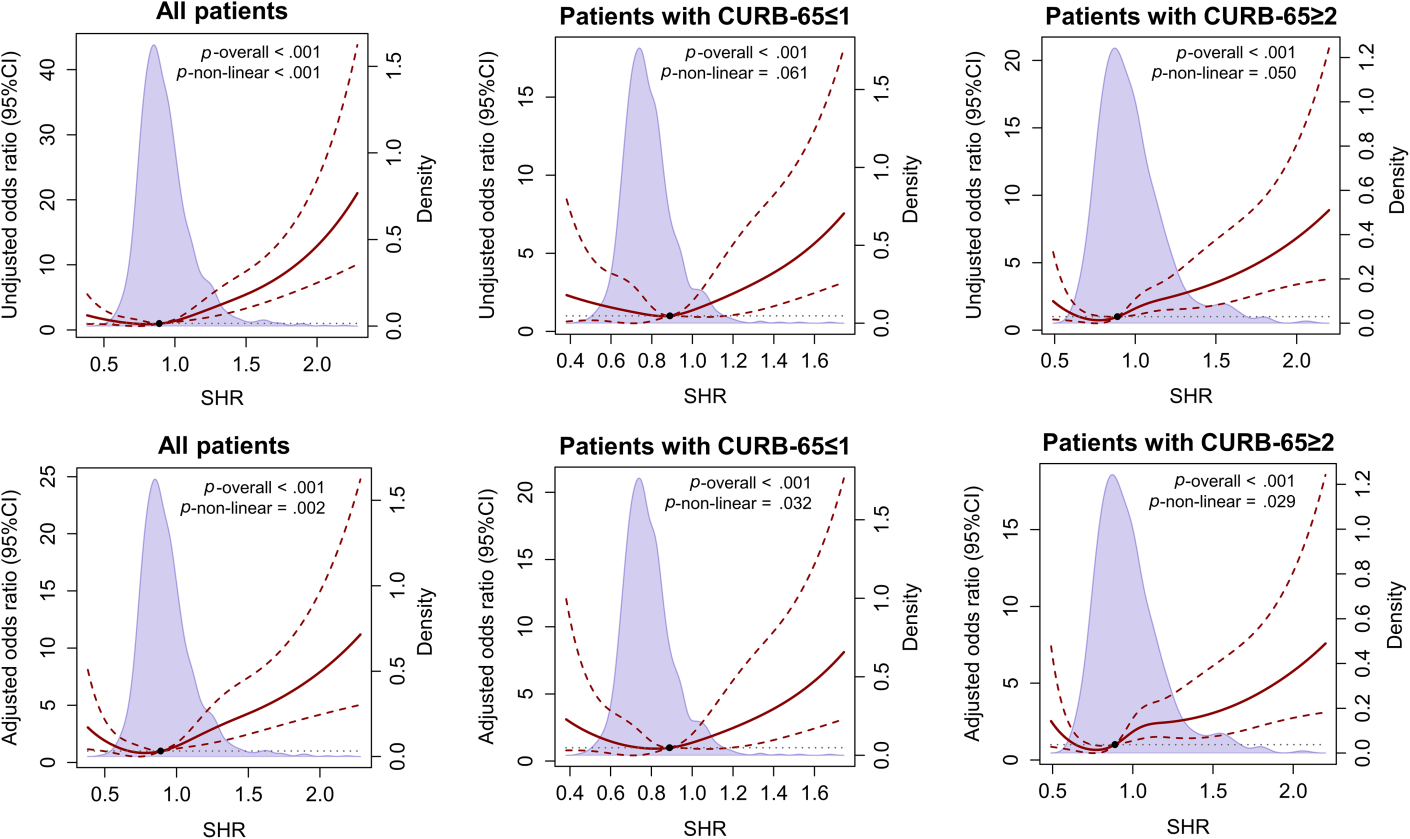
Restricted cubic splines showing unadjusted and adjusted odds ratios of SHR for adverse clinical outcomes in diabetic inpatients with pneumonia on admission. Odds ratios were analyzed in all patients (left column), patients with CURB‐65 ≤ 1 (middle column), and patients with CURB‐65 ≥ 2 (right column) separately by restricted cubic splines based on univariable and multivariable logistic regression analyses. In the multivariable logistic regression analyses, odds ratios were adjusted by age, sex, comorbidities (including chronic lung diseases, coronary heart disease, hypertension, stroke, chronic kidney disfunction, cirrhosis, rheumatic disease, and malignancy), current smoking status, and CURB‐65 scores on admission (as a continuous variable). Five knots for SHR were used both in the univariable and multivariable logistic regression analyses according to Akaike Information Criterion in general. Odds ratios were presented by the dark red solid lines and 95% CIs by the dark red dashed lines. Medians of SHR overall were indicated by the dark dot and set as a reference. Distributions of SHR in each group set were displayed by the light purple density plot. CI, confidence interval; CURB‐65, score for pneumonia severity (Confusion, blood Urea nitrogen, Respiratory rate, Blood pressure; SHR, stress hyperglycemia ratio.

Given the results of logistic regression analyses using SHR quartiles, we hypothesized that there was a nonlinear correlation between SHR and composite end point. Restricted cubic splines based on univariable and multivariable logistic regressions were explored to determine whether a nonlinear correlation existed or not. As displayed in Figure 3, nonlinear correlations were found in all patients in the univariable analyses (*p*‐nonlinear < .001) and in all groups in the multivariable analyses (*p*‐nonlinear = .002 in all patients; *p*‐nonlinear = .032 in patients with CURB‐65 ≤ 1; and *p*‐nonlinear = .029 in patients with CURB‐65 ≥ 2). In view of the fact that there was no commonly accepted threshold of low SHR, we roughly divided SHR into two parts based on median 0.889. In the univariable and multivariable logistic regression analyses, we found that when SHR was under median, risk of reaching composite endpoint significantly increased as SHR decreased; and when SHR was over median, risk of reaching composite endpoint significantly increased as SHR increased (Table 2).

**TABLE 2:** Unadjusted and adjusted odds ratios of SHR for adverse clinical outcomes in diabetic inpatients with pneumonia on admission.

Variable	Univariable logistic regression model		Multivariable logistic regression model	
	Odds ratio (95% CI)	*p* value	Adjusted odds ratio (95% CI)	*p* value
All patients				
SHR ≤ 0.889	0.09 (0.01–0.76)	.026	0.02 (0.00–0.84)	.040
SHR ≥ 0.889	8.31 (4.64–14.86)	.000	4.78 (2.53–9.05)	.000
Patients with CURB‐65 ≤ 1				
SHR ≤ 0.889	0.10 (0.01–1.86)	.122	0.03 (0.00–0.90)	.043
SHR ≥ 0.889	8.46 (2.98–24.01)	.000	9.53 (3.02–30.07)	.000
Patients with CURB‐65 ≥ 2				
SHR ≤ 0.889	0.06 (0.00–1.50)	.086	0.02 (0.00–0.84)	.040
SHR ≥ 0.889	4.31 (2.10–8.88)	.000	3.38 (1.58–7.22)	.002

*Note*: Statistical analyses were made by univariable and multivariable logistic regression. In the univariable logistic regression model, only SHR was included. In the multivariable logistic regression model, SHR was included along with age, sex, chronic comorbidities (including chronic lung diseases, coronary heart disease, hypertension, stroke, chronic kidney disfunction, cirrhosis, rheumatic disease, and malignancy), current smoking status, and CURB‐65 scores.

Abbreviations: CI, confidence interval; CURB‐65, score for pneumonia severity (Confusion, blood Urea nitrogen, Respiratory rate, Blood pressure); SHR, stress hyperglycemia ratio.

### 3.5 Predictive value of SHR for adverse clinical outcomes

Considering the nonlinear correlation between SHR and composite end point, we compared the predictive value of SHR as a spline term with that of SHR as a categorical variable (from Q1 to Q4) or as a continuous variable. In addition, we also compared the predictive value of SHR in different forms with FBG, which reflected the total blood glucose level with acute and routine blood glucose combined. Because FBG was linearly correlated with risk of reaching composite endpoint (*p*‐nonlinear = .373 in the univariable logistic regression model; *p*‐nonlinear = .480 in the multivariable logistic regression model), we took FBG as a continuous variable in the comparisons. As shown in Table 3, AUC was used to evaluate the predictive ability of SHR or FBG by univariable and multivariable logistic regression. In the univariable logistic regression model, AUC of SHR as a spline term was larger than that of SHR either as a categorical variable (0.699 vs 0.669, *p* = .002) or as a continuous variable (0.699 vs 0.665, *p* = .047) in all patients, and also larger than that of SHR as a categorical variable in patients with CURB‐65 ≥ 2 (0.678 vs 0.649, *p* = .041). In the multivariable logistic regression model, predictive value of the model containing SHR as a spline term was larger than that of the model containing SHR as a categorical variable in all patients (0.831 vs 0.822, *p* = .040). These results indicated the added predicted value of low SHR in the analysis of adverse clinical outcomes. As to the AUC difference between SHR and FBG, SHR in different forms all showed increased AUC in contrast with FBG (*p* < .05) in the univariable logistic regression model in all patients. In the multivariable logistic regression model, only the model containing SHR as a spline term possessed larger AUC than that of the model containing FBG (0.755 vs 0.722, *p* = .027).

**TABLE 3:** Predictive value of SHR and FBG for adverse clinical outcomes in diabetic inpatients with pneumonia on admission.

Variable	Univariable logistic regression model			Multivariable logistic regression model		
	AUC (95% CI)	*p* value^a^	*p* value^b^	AUC (95% CI)	*p* value^a^	*p* value^b^
All patients						
SHR (as a spline term)	0.699 (0.656–0.741)	1.000	<.001	0.831 (0.797–0.865)	1.000	.054
SHR (as a categorical variable)	0.669 (0.627–0.710)	.002	.021	0.822 (0.787–0.856)	.040	.560
SHR (as a continuous variable)	0.665 (0.616–0.714)	.047	.029	0.823 (0.788–0.858)	.117	.352
FBG (as a continuous variable)	0.624 (0.577–0.671)	<.001	1.000	0.818 (0.784–0.851)	.054	1.000
Patients with CURB‐65 ≤ 1						
SHR (as a spline term)	0.655 (0.581–0.730)	1.000	.131	0.792 (0.723–0.860)	1.000	.705
SHR (as a categorical variable)	0.618 (0.544–0.691)	.133	.453	0.774 (0.707–0.841)	.141	.454
SHR (as a continuous variable)	0.603 (0.516–0.690)	.200	.735	0.784 (0.716–0.851)	.518	.874
FBG (as a continuous variable)	0.590 (0.507–0.672)	.131	1.000	0.786 (0.721–0.850)	.705	1.000
Patients with CURB‐65 ≥ 2						
SHR (as a spline term)	0.678 (0.621–0.734)	1.000	.074	0.755 (0.703–0.807)	1.000	.027
SHR (as a categorical variable)	0.649 (0.596–0.702)	.041	.540	0.749 (0.696–0.801)	.530	.129
SHR (as a continuous variable)	0.654 (0.593–0.715)	.182	.367	0.736 (0.682–0.790)	.099	.221
FBG (as a continuous variable)	0.635 (0.575–0.695)	.074	1.000	0.722 (0.666–0.778)	.027	1.000

*Note*: In the univariable logistic regression model, only SHR or FBG was included. In the multivariable logistic regression model, SHR or FBG was included along with age, sex, chronic comorbidities (including chronic lung diseases, coronary heart disease, hypertension, stroke, chronic kidney disfunction, cirrhosis, rheumatic disease, and malignancy), current smoking status, and CURB‐65 scores.

Abbreviations: AUC, area under curve; CI, confidence interval; CURB‐65, score for pneumonia severity (Confusion, blood Urea nitrogen, Respiratory rate, Blood pressure); FBG, fasting blood glucose; SHR, stress hyperglycemia ratio.

^a^ Means *p* value in the comparison with SHR (as a spline term).

^b^ Means *p* value in the comparison with FBG (as a continuous variable).

## 4 DISCUSSION

Our study offered a clear and intuitionistic picture of the inflammatory responses and nonlinear association between SHR and adverse clinical outcomes in diabetic inpatients with pneumonia of different severity. Also, our study contained the largest number of patients with diabetes compared with studies of the same topic so far. The findings were as follows. Patients in the highest SHR quartile were of significantly elevated systematic inflammatory biomarkers, indicating inflammatory overreaction in these patients with diabetes. There were J‐shaped associations of SHR with adverse clinical outcomes in patients of different severity classified by CURB‐65 scores, even when those with absolute hypoglycemia were already excluded from the analyses. The hazard of low SHR was more obvious in patients with CURB‐65 ≥ 2 than those with CURB‐65 ≤ 1. These results suggested that FBG alone was not sufficient enough for the evaluation of insufficient blood sugar supply, especially in those with severer pneumonia. Predictive value of SHR for adverse clinical outcomes was greater than that of FBG and was further elevated when the nonlinear association was taken into consideration. Our study suggested the need to include stress hyperglycemia in the blood glucose management of diabetic inpatients with pneumonia, especially for the prevention of potential hypoglycemia and recognition of insufficient blood sugar supply in those of severe pneumonia or high HbA_1C_.

WBC, NLR, CRP, PCT, ESR, and D‐dimer are commonly used inflammatory biomarkers in pneumonia, reflecting the inflammation intensity or degree of lung injury.^26^ In face of stress and infection, complex interactions exist between stress hyperglycemia and inflammation.^27^ Briefly, stress hormones regulated by the hypothalamic–pituitary–adrenal (HPA) axis and the sympathoadrenal system profoundly modify the inflammatory response while raising blood glucose.^27^, ^28^ Glucose in itself is also a proinflammatory molecule, and inflammatory mediators can in turn downregulate glucose transporter‐1 and aggravate insulin resistant.^27^, ^28^ In our study, patients in Q4 showed markedly elevated level in these commonly used inflammatory indicators (Figure 1). These results suggested that patients in Q4 were under strong, or excessive, inflammatory response. Mondal et al found that level of CRP, lactate dehydrogenase, and D‐dimer were higher in coronavirus disease 2019 inpatients with SHR≥1.14.^14^ Data from Su et al also showed elevated neutrophil counts in patients admitted to intensive care unit (ICU) with higher GAR.^18^ Though hypoglycemia was also taken as a kind of stress, no difference was found in the level of inflammatory biomarkers between patients of Q1 (the lowest SHR) and those of Q2 or Q3 (the medium SHR), and so did it in previous studies of similar topics.^10^, ^17^, ^21^, ^27^ This may be explained by the exclusion of patients with absolute hypoglycemia on admission in our analyses and the relatively low severity of pneumonia in Q1 patients.

Associations of SHR with adverse clinical outcomes in diabetic patients with pneumonia were explored by logistic regressions, in which potential confounding factors with complete available data were taken into consideration. SHR was proven to be independently associated with adverse clinical outcomes in patients with different severity on admission in our analyses (as shown in Figures 2 and 3), in line with previous studies on pneumonia or other acute diseases.^13^, ^14^, ^15^, ^16^, ^17^, ^18^, ^19^, ^20^, ^21^ The strong association of excessive stress hyperglycemia and adverse clinical outcomes was quite obvious in our study and previous ones. Stress hyperglycemia is considered as an survival response to ensure an adequate glucose supply for non‐insulin dependent tissues.^10^ Generally, the HPA axis and the sympathoadrenal system will be activated to certain level according to the degree of stress,^10^ and severe stress hyperglycemia can lead to hypertonic state and cause fluid transfer, thus leading to renal impairment and volume depletion.^10^ According to the results of our study and previous ones, the harm of excessive stress hyperglycemia could also be partly explained by excessive inflammatory response.^14^, ^18^ Adequate blood glucose and inflammatory response are indispensable for fighting against pathogens.^29^ When blood glucose or inflammatory response was out of control, a vicious cycle could be set up.^27^

Several previous studies displayed the hazard of relative low stress hyperglycemia.^13^, ^16^, ^17^, ^21^ Among these three studies, one owas conducted on community acquired pneumonia (CAP) and two on critical illness. The study on CAP pointed out that patients with the lowest and highest quartiles of glycemic gap both had increased risk of 90‐day mortality in all patients after adjusted by status of diabetes, whereas no association was found when admission glycemic gap was taken as a continuous variable.^16^ We used restricted cubic splines for nonlinear analyses and made up for its shortcomings in the analysis with stress hyperglycemia as a continuous variable.The former study made the point that diabetic status had no influence on the relation between glycemic gap and 90‐day mortality.^16^ However, the author of a previous study in ICU pointed out that there appeared to be an elevated mortality in the lowest SHR group among patients with HbA_1C_ ≥ 6.5% but not among those with HbA_1C_ < 6.5%.^13^ Because HbA_1C_ level was quite different in people with and without diabetes, the association of SHR with clinical outcomes might be different in diabetic and nondiabetic people. Another study in ICU revealed a U‐shaped association between the glycemic gap and mortality.^17^ Here, we clearly displayed the ubiquitous J‐shaped relationships between SHR and adverse clinical outcomes in patients with pneumonia of different severity. Tthe hazard of Q1 was more obvious in patients with CUBR‐65 ≥ 2. Because CURB‐65 scores and inflammatory indicators of patients in Q1 were comparable with those in Q2, the increased risk of adverse clinical outcome might not be resulted from severer illness or stronger inflammatory response in our study, which was supposed as a probable reason in another study conducted among ICU patients.^17^ Because we noticed that Q1 patients had higher HbA_1C_ levels and higher rates of insulin usage for the routine hypoglycemic treatment, we inferred that patients in Q1 were a group of diabetic patients with poorer blood glucose control. Similarly, two previous studies, which found the J‐shaped or U‐shaped association between stress hyperglycemia and adverse clinical outcomes, both presented significantly higher HbA_1C_ level in patients with the lowest part of stress hyperglycemia.^17^, ^21^ It was reported previously that patients with high chronic premorbid hyperglycemia were of increased risk for hypoglycemia in ICU.^12^ So we supposed that the possible reason low SHR was associated with worse clinical outcomes could be the increased risk of developing hypoglycemia in the future.^7^ Risk of hypoglycemia is a major concern in the management of patients with diabetes in all guidelines.^5^, ^6^, ^7^, ^8^ Besides, in face of acute illnesses or injuries, graded responses to the degree of stress are needed and sufficient stress hyperglycemia is essential for immune and cerebral function.^10^, ^27^ So, another possible reason might be the relatively glucose deficient in these people. Glycemic management in diabetic inpatients should be modified according to previous blood glucose level and disease severity. A recent CONTROLING study reported that there was no survival benefit in ICU patients when blood glucose of patients was controlled to preadmission level compared with <180 mg/dL.^30^ As suggested in that study, further studies targeting a glycemia over the usual, which matched the stress‐induced hyperglycemia, were needed to test the benefit of individualized blood glucose control in inpatients.^30^ Indexes that can reflect stress‐induced hyperglycemia, SHR for instance, can well fit this need. According to restricted cubic splines based on logistic regression analyses, there was a level of SHR with the lowest OR of adverse clinical outcomes in diabetic inpatients with pneumonia. The nadir value might give insight into the individualized blood glucose management in diabetic inpatients with pneumonia.

What is more, SHR was considered a better predictive factor for adverse clinical outcomes in acute illness or injuries compared with blood glucose.^14^, ^15^, ^18^ Without the commonly accepted threshold of low SHR, we used SHR as a spline term, as quartiles, and a continuous variable to analyze its predictive value (Table 3). We found that the predictive value of SHR was improved when taking the J‐shaped association into consideration, especially in patients with more severe pneumonia. These results suggested that there seemed to be a range, with both lower and upper limits, of SHR that was associated with favorable clinical outcomes in diabetic patients with pneumonia on admission.

It was indicated in a previous study that the distribution of a risk factor in the study population greatly influenced its nadir value in a U‐ or J‐shaped relationship between a particular risk factor and a future health outcome, as well as the exact hazard ratio or OR.^31^ Consequently, considering the sample size of this study, we did not point out the nadir value or make further investigation on the exact range. Further large‐scale studies are needed to find this nadir value and favorable range of SHR and make them better serve in the prediction of adverse clinical outcomes in diabetic inpatients with pneumonia.

There are certain limitations in the present study. First, data were retrospectively collected from medical records and missing data of certain degree existed, especially on HbA_1C_ and CURB‐65 scores. The sample size and the data incompleteness made it hard to address other potential confounding factors in this study, such as body mass index, vaccination status, insulin treatment, diabetes‐related complications, etc. Further prospective researches are needed to remedy this shortcoming. Second, CURB‐65 score is a simple but rough evaluation method of pneumonia severity. Third, we presented only the J‐shaped association of SHR with adverse clinical outcomes but did not provide the exact cutoff points of SHR and other evaluation indicators in the prediction model except AUC. Fourth, restricted by the low positive rate of pathogen detection, limited number of participants with type 1 diabetes, and small number of patients with adverse clinical outcomes, we needed to use a composite end point in the prognostic analysis, as in a previous study^32^ and did not verify the applicability of our findings with regard to cause of pneumonia, type of diabetes, and separate adverse clinical outcome. Further studies with larger sample sizes or clinical trials are needed to verify the benefits of SHR, or other forms of stress‐induced glycemia, in the individualized management in diabetic patients with pneumonia or other acute illnesses.

In conclusion, we found that high SHR was associated with elevated systematic inflammation in diabetic inpatients with pneumonia on admission. J‐shaped associations of SHR with adverse clinical outcomes commonly existed in diabetic inpatients with pneumonia of different severity. The inclusion of SHR in the blood glucose management of patients with diabetes may be beneficial, especially for the prevention of potential hypoglycemia or the recognition of glucose insufficient in those of severe pneumonia or high HbA_1C_.

## AUTHOR CONTRIBUTIONS

Min Zhou conceived and designed the study. Bing Liu, Yu Chen, and Liping Yu collected and analyzed the data. Bing Liu drafted the manuscript. Min Zhou revised the manuscript. All authors read and gave final approval of the version to be submitted.

## FUNDING INFORMATION

This work was supported by the National Key R&D Program of China (Grant Nos. 2017YFC1309700 and 2017YFC1309701), Shanghai Municipal Key Clinical Specialty (shslczdzk02202), Shanghai Top‐Priority Clinical Key Disciplines Construction Project (2017ZZ02014), Shanghai Key Laboratory of Emergency Prevention, Diagnosis and Treatment of Respiratory Infectious Diseases (20dz2261100), and Cultivation Project of Shanghai Major Infectious Disease Research Base (20dz2210500).

## CONFLICT OF INTEREST STATEMENT

The authors declare no conflicts of interest in this work.

## Supporting information


**Figure S1.** Flow chat of the study. SHR, stress hyperglycemia ratio.
