# Supplementary figures and images for: Stress hyperglycemia ratio is associated with systemic inflammation and clinical outcomes in diabetic inpatients with pneumonia on admission
Source: J Diabetes. 2023 May 5;15(7):545–56. doi: 10.1111/1753-0407.13398 (PMC10345973; doi:10.1111/1753-0407.13398)

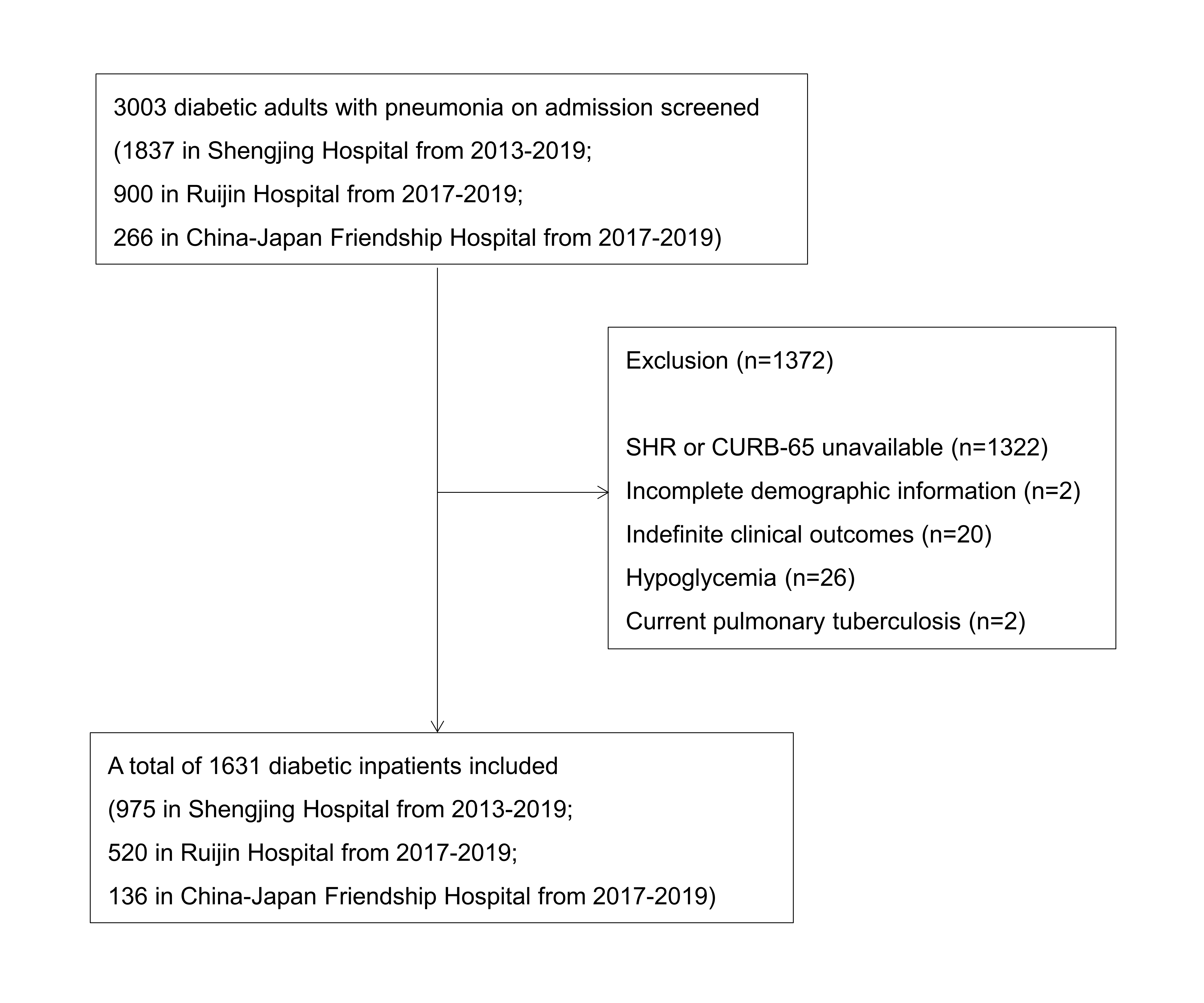

Supplement: Supplementary file 1 — Figure S1. Flow chat of the study. SHR, stress hyperglycemia ratio. [file JDB-15-545-s001.tif]
